# Aerobic exercise induces tumor suppressor p16^INK4a^ expression of endothelial progenitor cells in human skeletal muscle

**DOI:** 10.18632/aging.103763

**Published:** 2020-10-26

**Authors:** Jinfu Wu, I-Shiung Cheng, Suchada Saovieng, Wei-Horng Jean, Chung-Lan Kao, Yung-Yang Liu, Chih-Yang Huang, Tania Xu Yar Lee, John L. Ivy, Chia-Hua Kuo

**Affiliations:** 1 Laboratory of Regenerative Medicine in Sports Science, School of Physical Education and Sports Science, South China Normal University, Guangzhou, China; 2 Laboratory of Exercise Biochemistry, University of Taipei, Taipei, Taiwan; 3 Laboratory of Exercise Nutrition, National Taichung University of Education, Taichung, Taiwan; 4 Department of Anesthesiology, Far East Memorial Hospital, New Taipei, Taiwan; 5 Department of Physical Medicine and Rehabilitation, Taipei Veterans General Hospital and National Yang Ming University, Taipei, Taiwan; 6 Department of Chest Medicine, Taipei Veterans General Hospital and National Yang Ming University, Taipei, Taiwan; 7 Chinese Medicine, Hualien Tzu Chi Hospital, Tzu Chi Medical Foundation, Tzu Chi University, Hualien, Taiwan; 8 College of Sports Science and Technology, Mahidol University, Bangkok, Thailand; 9 Department of Kinesiology and Health Education, The University of Texas at Austin, TX 78712, USA

**Keywords:** cancer, tumor, cell cycle arrest, skeletal muscle

## Abstract

Aerobic exercise induces oxidative stress and DNA damage, nevertheless, lowers cancer incidence. It remains unclear how genetic stability is maintained under this condition. Here, we examined the dynamic change of the tumor suppressor p16^INK4a^ in cells of skeletal muscle among young men following 60-min of aerobic cycling at 70% maximal oxygen consumption (V̇O_2max_). Rg1 (5 mg, an immunostimulant ginsenoside) and placebo (PLA) were supplemented 1 h before exercise. Data from serial muscle biopsies shows unchanged p16^INK4a+^ cells after exercise followed by a considerable increase (+21-fold) in vastus lateralis muscle 3 h later. This increase was due to the accumulation of endothelial progenitor cells (p16^INK4a+^/CD34^+^) surrounding myofibers and other infiltrated nucleated cells (p16^INK4a+^/CD34^-^) in necrotic myofibers. During the Rg1 trial, acute increases of p16^INK4a+^ cells in the muscle occurred immediately after exercise (+3-fold) and reversed near baseline 3 h later. Rg1 also lowered IL-10 mRNA relative to PLA 3 h after exercise. Post-exercise increases in VEGF mRNA and CD163^+^ macrophages were similar for PLA and Rg1 trials. Conclusion: The marked increases in p16^INK4a^ protein expression of endothelial progenitor cells in skeletal muscle implicates a protective mechanism for maintaining genetic stability against aerobic exercise. Rg1 accelerates resolution of the exercise-induced stress response.

## INTRODUCTION

Most of the cells within the human body are short-lived and age rapidly [1], where birth, aging, and death of cells are continuously occurring to sustain a stable cell population. Capillary endothelial cells have an average lifespan ranging from 2 to 15 days in mammalian tissues [1, 2]. Endothelial progenitor cells (CD34^+^) from bone marrow are responsible for the rapid replacement of unhealthy endothelial cells [3], as well as the donation of their nuclei to injured myofibers for regeneration [4]. We have recently reported various amounts of p16^INK4a+^ endothelial progenitor cells in more than 40% of capillaries surrounding myofibers in human skeletal muscle of young men between 20-25 years of age [5].

Given a huge amount of daily cell turnover in human adults, a random genetic mutation is unavoidable following numerous cell division. The protein p16^INK4a^, known as a tumor suppressor, is a protein inhibitor of the DNA synthesis phase of the cell cycle expressed in replicable cells [6]. Both cellular senescence and stress elevate p16^INK4a^ protein in replicable cells, which induces cell cycle arrest [6, 7], and illustrates the protective role of p16^INK4a^ for the fidelity during cell division in large multicellular systems. Downregulation of p16^INK4a^ has been observed in a large number of tumors in humans.

Aerobic exercise transiently increases oxidative stress and DNA damage [8]. However, participating aerobic exercise is associated with lower cancer incidence in both animals and humans [9]. The underlying mechanism remains unclear. In this study, distribution of p16^INK4a+^ endothelial progenitor cells in vastus lateralis muscle were examined in young adults. Induction of cellular senescence facilitates immune clearance of unhealthy replicable cells by activating immune responses [10]. To further investigate the role of the immune system on dynamical changes of p16^INK4a+^ senescent cells, Rg1 was orally ingested 1 h before exercise [11, 12]. Rg1 is an immunostimulant during inflammation, which activates macrophage function [11, 13].

## RESULTS

**Figure 1:**
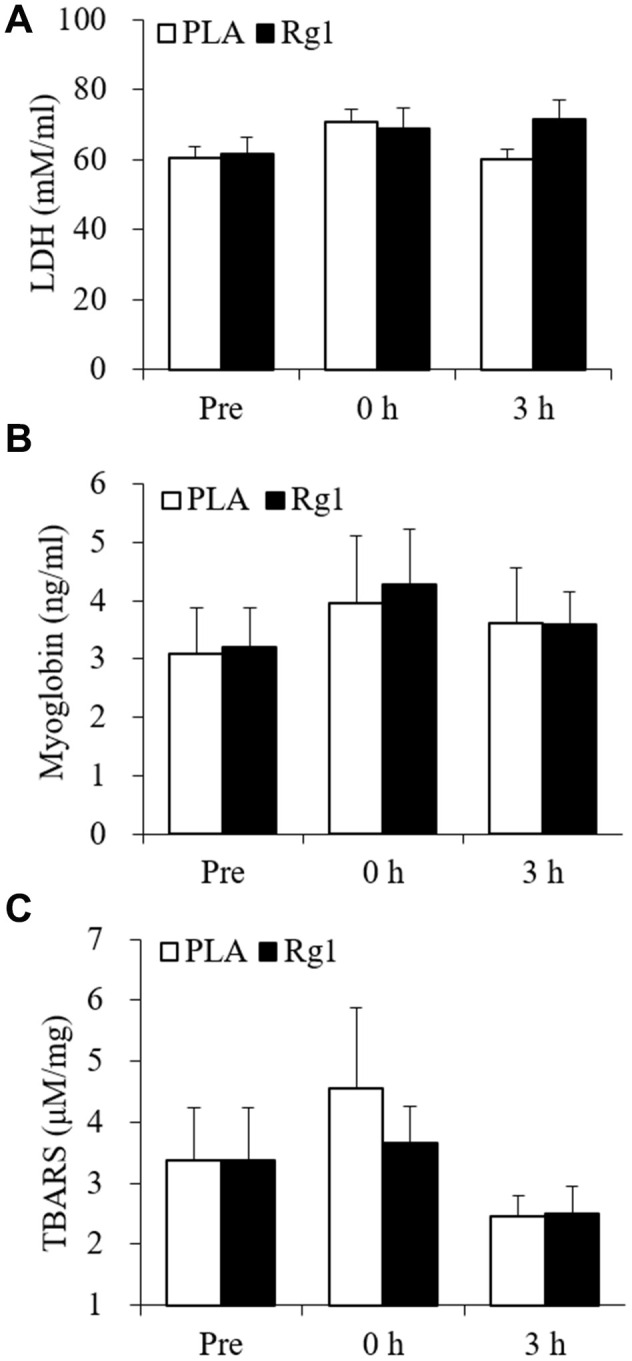
**Plasma muscle damage markers unaltered following aerobic exercise.** Lactate dehydrogenase (**A**), myoglobin (**B**), and TBARS (**C**) were not significantly increased after 1-h cycling exercise (70% V̇O_2max_). Data are expressed as mean and SEM. Abbreviation: TBARS, Thiobarbituric acid reactive substances.

**Figure 2:**
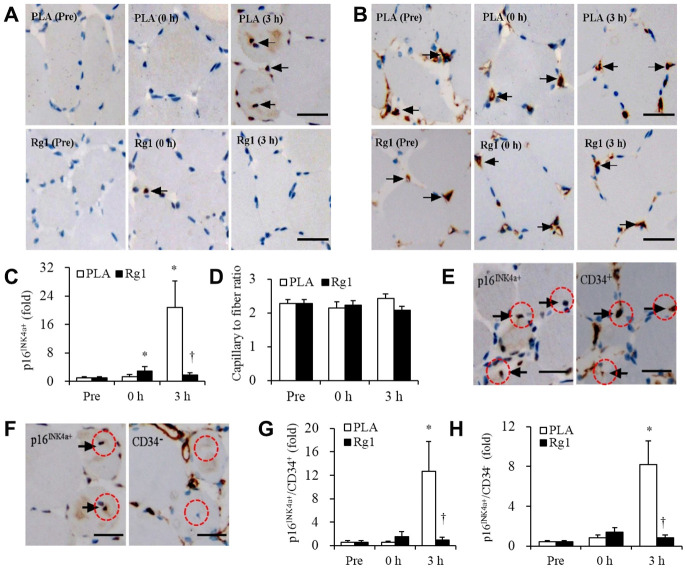
**Massive increases of senescent endothelial progenitor cells in human skeletal muscle 3 h after aerobic exercise (70% V̇O_2max_).** Approximately 21-fold increases in senescent endothelial progenitor cells occurred 3 h after 1-h cycling exercise (70% V̇O_2max_), while Rg1 supplementation advances the increase immediately after exercise (~3-fold) and decline to baseline 3 h after exercise (**A**, **C**). Total endothelial progenitor cells (CD34^+^) surrounding myofibers were unaltered after exercise for both trials (**B**, **D**). Approximately 60% of elevated senescent endothelial progenitor cells was contributed by endothelial progenitor cells (p16^INK4a+^ / CD34^+^) (**E**, **G**), while the rest of 40% was contributed by infiltrated nucleated cells (p16^INK4a+^ / CD34^-^) (**F**, **H**). Scale bar = 30 μm. Data are expressed as mean and SEM. * Significant difference against Pre (Baseline), P < 0.01; † Significant difference against Placebo, P < 0.01. Abbreviation: PLA, Placebo.

**Figure 3:**
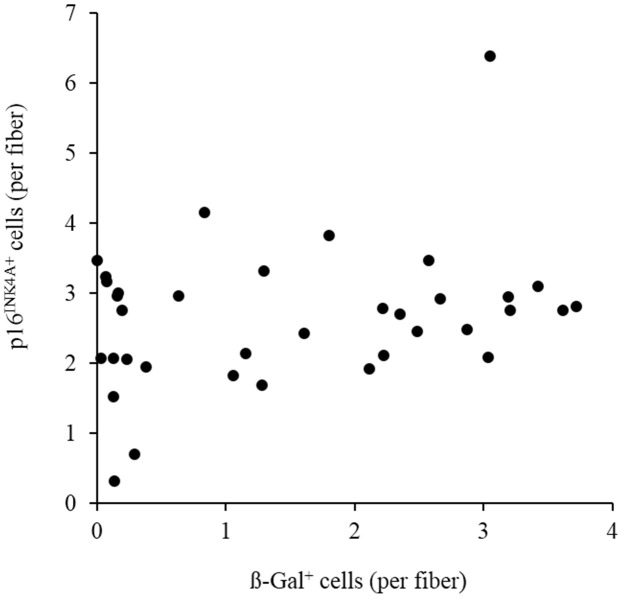
**Moderate correlation (r = 0.29, p = 0.08) between p16^INK4a^ positive cells and ß-galactosidase positive cells of 36 biopsied vastus lateralis muscle in men aged 20-26 y.** Abbreviation: ß-gal, ß-galactosidase.

Endurance cycling at 70% V̇O_2max_ for 1 h does not produce significant increases in circulating LDH and myoglobin (Figure 1). The lipid peroxidation marker TBARS levels tends to increase by 35% during the PLA trial (Figure 1C). No detectable change in p16^INK4a+^ senescent cells was observed immediately after exercise (PLA trial). However, ~21-fold increases (P < 0.01) in p16^INK4a+^ senescent cells of skeletal muscle occurred 3 h after exercise. During the Rg1 trial, ~3-fold increases (P < 0.05) in p16^INK4a+^ senescent cells of skeletal muscle were observed immediately exercise followed by ~40% decline 3 h after exercise (P < 0.05) (Figure 2A, 2C). CD34^+^ cell-to-fiber ratio of skeletal muscle was not altered after exercise (Figure 2B, 2D). A great portion of senescent cell accumulation in exercised muscle is contributed by increased senescent endothelial progenitor cells (p16^INK4a+^/CD34^+^) (Figure 2E, 2G). Approximately 40% of the increases is associated with other types of nucleated cells (p16^INK4a+^/CD34^-^) localized mostly in necrotic myofibers (Figure 2F, 2H). Dramatic increases in p16^INK4a+^ cells 3 h after exercise is potently lowered by Rg1 supplementation (P < 0.05). A moderate correlation (r = 0.29, p = 0.08) was found between p16^INK4a^ positive cells and ß-galactosidase positive cells of 36 biopsied muscle samples in men aged 20-26 y (Figure 3).

**Figure 4:**
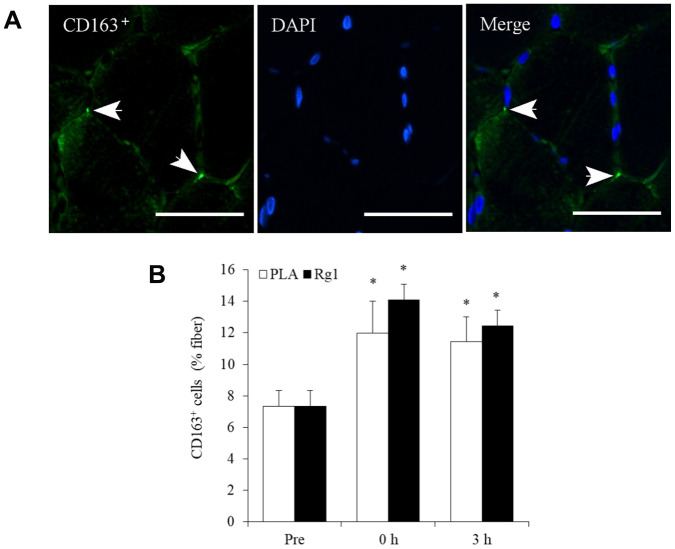
**Aerobic exercise increases regenerative macrophage infiltration into human skeletal muscle.** Arrows in the representative images indicate CD163^+^ cells (bright green) and nuclei (blue) surrounding myofibers in a muscle cross-section (**A**). This increase after 1-h cycling exercise (70% V̇O_2max_) was similar for both Placebo (PLA) and Rg1 trials (**B**). Scale bar = 50 μm. Data are expressed as mean and SEM. * Significant difference against Pre (Baseline), P < 0.01. Abbreviation: PLA, Placebo.

Aerobic cycling (70% V̇O_2max_) increased CD163^+^ macrophage infiltration into human skeletal muscle (Figure 4). Placebo (PLA) and Rg1 trials show a similar magnitude of cell infiltration (PLA: 0 h, +63%, P < 0.05; 3 h, +56%, P < 0.05; Rg1: 0 h, +92%, P < 0.01; 3 h, +70%, P < 0.01).

**Figure 5:**
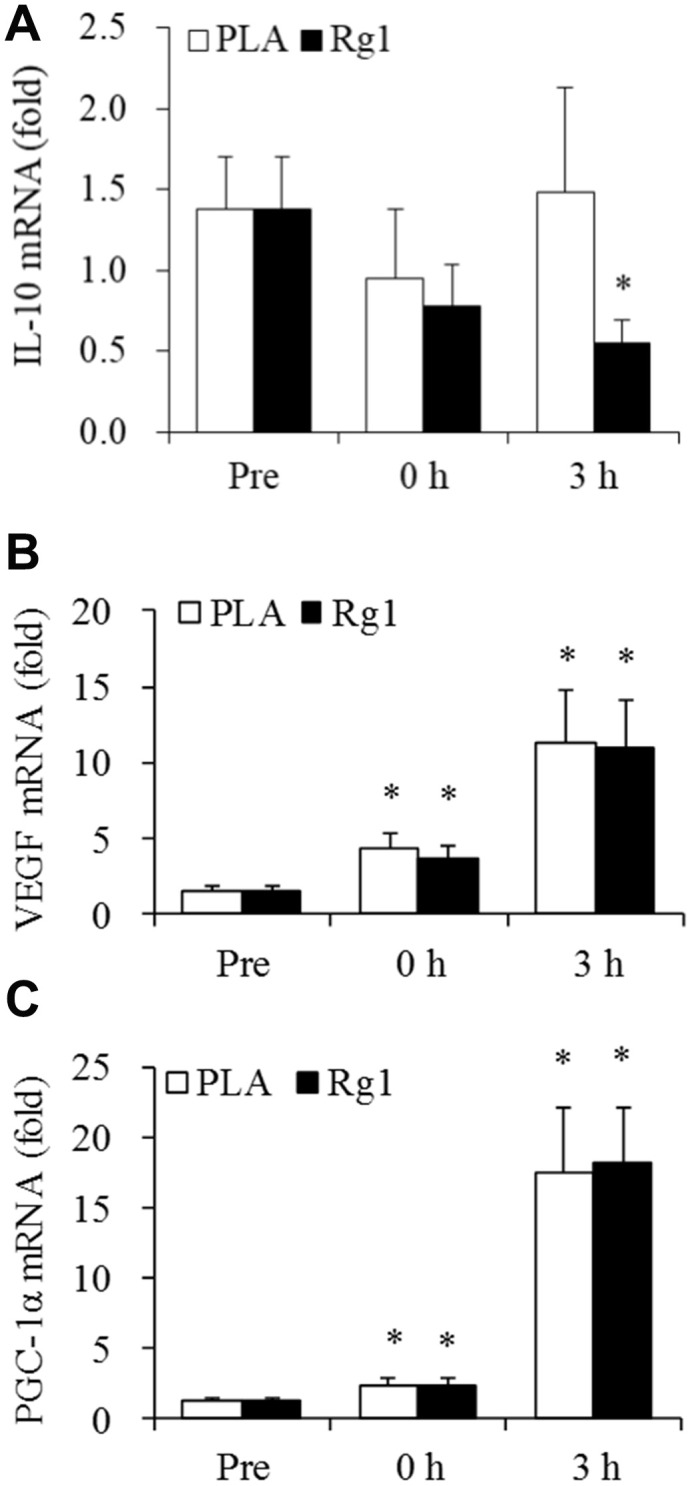
**IL-10, VEGF, and PGC1-alpha expression after aerobic exercise.** (**A**) Rg1 supplementation lowers IL-10 mRNA 3 h after exercise (-60%, P < 0.05). Dramatic elevations for VEGF mRNA (**B**) and PGC1-alpha mRNA (**C**) occurred 3 h after 1-h cycling exercise (70% V̇O_2max_). Data are expressed as mean and SEM. * Significant difference against Pre (Baseline), P < 0.05. Abbreviation: PLA, Placebo.

Data for exercise response in IL-10 mRNA, VEGF mRNA and PGC-1α mRNA of skeletal muscle are shown in Figure 5. IL-10 mRNA did not change after an acute bout aerobic exercise, while Rg1 significantly decreased IL-10 mRNA expression 3 h after exercise (-60%, P < 0.05) (Figure 5A). Both VEGF mRNA (Figure 5B) and PGC-1α mRNA (Figure 5C) increased significantly in challenged skeletal muscle. Similar increases were observed in VEGF mRNA of skeletal muscle for both PLA (0 h, +2-fold, P < 0.01; 3 h, +7-fold, P < 0.01) and Rg1 (0 h, +1-fold, P < 0.05; 3 h, +7-fold, P < 0.01) trials following exercise. PGC-1α mRNA also shows similar increases for PLA (0 h, +1-fold, P < 0.05; 3 h, +13-fold, P < 0.01) and Rg1 (0 h, +1-fold, P < 0.01; 3 h, +14-fold, P < 0.01) trials.

## DISCUSSION

Aerobic exercise induces VEGF expression and stimulates angiogenesis [14]. During this process, endothelial progenitor cells are capable of self-replicating to increase cell number to renew damaged endothelial cells in the capillaries [15]. However, oxidative DNA damage is increased after an acute bout of aerobic exercise [8, 16]. In this study, we found a considerable rise in the tumor suppressor p16^INK4a^ protein expression in endothelial progenitor cells of human skeletal muscle after an acute bout of aerobic exercise (70% V̇O_2max)_. Furthermore, the immune-stimulant Rg1 accelerated the resolution of this stress response, evidenced by an earlier rise and fall of p16^INK4a+^ cell number in exercised muscle. Stress that causes DNA damage results in the increased expression of the p16^INK4a^ protein, which is responsible for inhibition of cell division [6]. Thus, the transient increases in p16^INK4a+^ protein expression may mirror the magnitude of stress-related DNA damage [16] and suggests a protective mechanism for maintaining genetic stability of replicable cells against aerobic exercise.

Increasing p16^INK4a^ protein expression is also known to stimulate tissue repair during inflammation [7, 17]. Inflammation is essential for recognition and phagocytic clearance of unhealthy cells that develops a senescence phenotype [18–20] followed by a protracted period of cell regeneration [21]. A recent study has reported an enhanced regenerative process after increasing p16^INK4a+^ mesenchymal stem cells during muscle inflammation [22]. Taken together, these recent findings suggest a new role of stress-induced p16^INK4a^ protein expression in inflammation-mediated muscle regeneration. Rg1 is an immunostimulant that activates macrophage [11] and advances the progression of inflammation from M1 to M2 phenotype in exercised human skeletal muscle [23]. Therefore, pre-exercise Rg1 intake is likely to potentiate the inflammation response (preconditioning) and result in an early resolution of the exercise stress response.

M2 macrophage (CD163^+^) polarization occurs during the later period of inflammation, which is responsible for cell regeneration of muscle tissue after physical challenge [21, 24]. However, massive increases in p16^INK4a+^ endothelial progenitor cells observed in the study was not directly associated with an exercise-induced increment of M2 macrophage in skeletal muscle. The magnitude of increase in M2 macrophage was similar for PLA and Rg1 trials, while the exercise-induced response of p16^INK4a+^ cells in human skeletal muscle was lower than that in the PLA trial. However, our results do not preclude the possibility that accumulation of p16^INK4a+^ endothelial progenitor cells directly increase M2 macrophage activity in human skeletal muscle.

A limitation of the study is the difficulty in determining whether increased p16^INK4a+^ cells 3 h post-exercise was completely attributed cell senescence or simply due to a reversible induction of p16^INK4a^ protein expression of well-functioned endothelial progenitor cells. Cellular senescence is featured by an irreversible form of cell-cycle arrest after prolonged stress. It is not possible to determine whether p16^INK4a+^ cell number is representing of irreversible cell-cycle arrest in human muscle tissue, since both p16^INK4a+^ cell accumulation and p16^INK4a+^ cell removal (immune clearance) can occur in the same muscle tissue following exercise. In this study, a moderate correlation between p16^INK4a^ positive cells and ß-galactosidase positive cells in 36 muscle samples suggests that p16^INK4a^ is not a perfect tissue senescence marker. This is in agreement with a previous study [25]. Whether p16^INK4a^ is a reliable cell senescence marker in human tissues cannot be settled in this study.

Another limitation is inadequate time points for muscle biopsies, which prevented us from delineating the timings of the rise-and-fall pattern for p16^INK4a+^ cell accumulation in exercising muscle. The acute response of p16^INK4a^ protein expression in endothelial cells of skeletal muscle after aerobic exercise is in sharp contrast to what has been observed after resistance exercise. We and others have previously shown decreased p16^INK4a+^ cells in muscle tissue of untrained active women and men after resistance training [5, 26]. Time required for resolution of the stress response and inflammation during and after exercise is associated with the magnitude of tissue damage, and varying by mode, intensity and duration of exercise. Furthermore, the local tissue response is influenced by distribution of the whole-body immune resource (white blood cells and stem cells from bone marrow). For example, aerobic exercise exerts a major challenge to the cardiopulmonary system in addition to skeletal muscle. The majority (> 60%) of white blood cells and stem cells are harbored in the lungs for constant local repair and regeneration [27, 28]. Aerobic exercise creates a competition between the lungs and muscle for immune resources for cell turnover [27]. In contrast, resistance exercise generates little challenge to the lungs, yet eccentric muscle contractions induce a substantial amount of muscle damage, which attracts more immune cells compared with aerobic exercise. Therefore, infiltration of immune cells into skeletal muscle after resistance exercise would be less likely to be compromised by competition with the lungs as occurs with aerobic exercise. Such differences can produce distinct rise-and-fall patterns for p16^INK4a+^ cells in human skeletal muscle [19, 29], and possibly explain the delayed response of p16^INK4a+^ cells in human skeletal muscle after aerobic exercise.

## CONCLUSIONS

We observed a considerable increase in p16^INK4a^ protein expression of endothelial progenitor cells in human skeletal muscle 3 h after aerobic exercise at 70% V̇O_2max_. The result of the study suggests that increased p16^INK4a^ expression is a protective mechanism to maintain genetic stability of replicable cells during regenerative phase after aerobic stress. Early resolution of this stress response occurs when the immunostimulant Rg1 is orally taken 1 h before exercise.

## MATERIALS AND METHODS

### Participants

Twelve healthy young men 20-23 years of age (weight: 51-95 kg; height: 161-190 cm) with V̇O_2max_ ranging between 43-55 ml^−1^ kg^−1^ min^−1^ volunteered to participate in this study. Participants were untrained and recreationally active non-smokers. They were fully informed of the risks and discomfort associated with the study, and all provided written consent before participation. This study was conducted in accordance with the guidelines contained in the Declaration of Helsinki and was approved by the Institutional Review Board of University of Taipei, Taipei, Taiwan.

### Experimental design

A placebo-controlled, counter-balanced, crossover study was conducted. Participants were randomized into one of two groups: PLA (5 mg of cellulose) and Rg1 (5 mg). Rg1 was obtained from NuLiv Science, Inc. (Brea, CA, USA). Participants randomly assigned to the PLA group received cellulose before trial one, and Rg1 before trial two. Accordingly, participants randomly assigned to the Rg1 group received Rg1 before trial one and cellulose before trial two. Trials one and two were separated by ten days. The Rg1 and cellulose were provided in capsules 1 h before exercise. Capsules were coded by number for later identification. Exercise consisted of 1 h of continuous cycling at 70% V̇O_2max_ on a cycle ergometer (Monark 839E, Stockholm, Sweden).

### Experimental protocol

All participants were familiarized with the experimental procedures and equipment before testing started. Participants completed a V̇O_2max_ test using a graded exercise protocol on a cycle ergometer one week before starting the experimental trials. All participants consumed a standard isocaloric diet 12 h prior to each experimental trial to limit any potential dietary effect that could influence the outcome of the study. Participants orally ingested 5 mg of Rg1 or PLA 1 h before cycling. After exercise, participants consumed a high carbohydrate meal (1.5 g carbohydrates per kg body weight: carbohydrate 80%, fat 8% and protein 12%; glycemic index: 80) within 10 min at the start of a 3-h recovery period. Water was provided ad libitum during and after the meal.

### Muscle biopsy

Muscle samples were taken from vastus lateralis muscle before (Pre), immediately (0 h) and 3 h after the 1-h cycling exercise protocol. Biopsies were performed under local anesthesia (2% lidocaine) by a certified physician using an 18G Temno disposable cutting needle (Cardinal Health, Waukegan, IL, USA). Biopsies were taken from the vastus lateralis 3 cm in depth and 20 cm proximal to the knee. The baseline muscle biopsy (Pre) was conducted 3-4 weeks before the start of the first 1 h cycling exercise test. Two consecutive muscle biopsies were performed immediately (0 h) after and 3 h after each cycling test. The 0 h and 3 h biopsies were taken from opposite legs, but at the same position on the vastus lateralis. A portion of each muscle sample was immediately frozen in liquid nitrogen and stored at -70 °C until analyzed. The rest of the sample was immediately placed in a conical vial containing 10% formalin and used for immunohistochemical analysis. Paraffin-embedded tissue was sectioned no later than 3 h following muscle sample collection.

### Immunohistochemistry

Serial sections of paraffin-embedded tissue were cut and analyzed for distribution of p16INK4a and CD34 in the vastus lateralis muscle. Paraffin sections (8 μm thick) were labeled using immunohistochemistry for binding of human monoclonal antibody p16^INK4a^ (1:200, ab108349; Abcam, Cambridge, MA, USA) and CD34 (1:200, ab81289; Abcam, Cambridge, MA, USA). Immunofluorescence was used to detect regenerative macrophage CD163 (1:400, ab87099; Abcam, Cambridge, MA, USA) infiltration in the vastus lateralis after exercise. Optical images were analyzed using ImageJ (NIH, Bethesda, MD). Positive markers within cells were quantified and expressed as positive signal number/total skeletal muscle fiber number (%). An average of 600 muscle fibers per slide were included for analysis. All analyses were conducted by a specialist at the University of Taipei and a certified pathologist from Taipei Institute of Pathology with similar results. An additional 36 muscle biopsied samples were used to assess correlation between p16^INK4a^ positive cells and ß-galactosidase positive cells (1: 150, NBP2-45731, Novus Biologicals, CO, USA).

### Quantitative PCR

RNA was extracted from ~15 mg of skeletal muscle using TRI Reagent (T9424-200) (Sigma, St. Louis, MO, USA) for homogenization, followed by isopropanol precipitation, two ethanol washes, drying, and suspension in 20 μl nuclease-free water. One microgram of RNA in a total volume of 20 μl was reverse transcribed to cDNA using iScript cDNA Synthesis Kit (#170-8890) (Bio-Rad, Hercules, CA, USA). Real-time PCR was performed using MyiQ Single Color Real-Time PCR Detection System (Bio-Rad, Hercules, CA, USA), TaqMan Probe (Sigma-Aldrich, Singapore) and iQ Supermix kit (#170-8860) (Bio-Rad, Hercules, CA, USA). The PCR conditions for all genes consisted of one denaturing cycle at 90°C for 30 s, annealing at 60°C for 60 s and elongation at 72°C for 60 s. At the end of the PCR the samples were subjected to a melting curve analysis. To control for any variations due to efficiencies of the reverse transcription and PCR, 18S ribosomal RNA was used as an internal standard to determine relative expression levels of the target mRNAs. The primers and probes used to amplify target mRNA are 18S ribosomal (18S): Forward (5’-3’): ACAGGATTGACAGATTGATAGCTC, Reverse (5’-3’): TCGCTCCACCAACTAAGAACG, Probe (5’-3’): TGCACCACCACCCACGGAATCGAG; IL-10: Forward (5’-3’): CTTCCCTGTGAAAACAAG, Reverse (5’-3’): AGACCTCTAATTTATGTCCTA, Probe (5’-3’): AGTCGCCACCCTGATGTCTC; VEGF: Forward (5’-3’): TGAGATCGAGTACATCTTCAAGCC, Reverse (5’-3’): GGCCTTGGTGAGGTTTGATCC, Probe (5’-3’): CCTGTGTGCCCCTGATGCGATGCG; PGC-1α: Forward (5’-3’): CGAGGAATATCAGCACGAGAGG, Reverse (5’-3’): CATAAATCACACGGCGCTCTTC, Probe (5’-3’): TGCCTTCTGCCTCTGCCTCTCCCTC.

### Serum LDH, Myoglobin and TBARS

A colorimetric assay kit was used to detect serum LDH activity according to the manufacturer’s instructions (Bio-Vision, # k726-500, CA, USA). Myoglobin was measured by ELISA using a commercially available kit (Immunology Consultants Laboratory, E-80MY, OR, USA). Serum samples were also used after further dilution for measurement of TBARS using a commercially available kit (Cayman Chemical, No. 10009055, MI, USA).

### Statistical analysis

All data are expressed as means ± standard error. The data were analyzed using a two-factor repeated-measures ANOVA (SPSS 20.0). Post hoc paired comparison analysis was performed with the Fisher LSD method. Type I error of P ≤ 0.05 was considered significant. P ≤ 0.1 was considered moderately significant.
